# A Novel Protein Serum Biomarker Assay for Tracking (Neo)adjuvant and Metastatic Therapy Efficacy and Enabling the Timely Detection of Relapse in Breast Cancer

**DOI:** 10.3390/cancers17244004

**Published:** 2025-12-16

**Authors:** Jeffrey Dea, Christine Chavany, Rosaura P. C. Valle, Rafael Hernández González, Moncef Jendoubi

**Affiliations:** 1 Milagen, Inc., Emeryville, CA 94608, USA; 2 Instituto Nacional de Ciencias Médicas y Nutrición Salvador Zubirán, Tlalpan 14080, Ciudad de México, CDMX, Mexico; rafa.hernandez@unam.mx

**Keywords:** breast cancer, biomarker, immunoassay, diagnosis, monitoring, surveillance

## Abstract

**Objectives**: There is high interest in blood-based assays for tracking treatment efficacy and detecting recurrence among breast cancer patients. This preliminary feasibility study assessed the performance of the Early Detection Serum-based Assay for Breast Cancer (EDSA-BC), which measures a novel protein biomarker (BF-09), in (neo)adjuvant and metastatic treatment follow-up. **Methods**: In total, 313 serial serum samples collected retrospectively from 72 BC patients (72.2% stage I–III, 27.8% stage IV) during disease management (median 33.4 months) were analyzed for BF-09 serum level at different time points before and/or after surgery and treatment (3 to 9 samples per patient). Serial dynamics of BF-09 were correlated with clinically recorded outcomes. **Results:** Per-sample performance of EDSA-BC for progressive disease or recurrence was 74% sensitivity at 85% specificity with 49.4% positive predictive value, 94.3% negative predictive value and 83% accuracy. Median lead time in detecting relapse was 9.4 months (range 0.8–27 months). **Conclusions**: Results showed the clinical validity of BF-09 in reflecting relative tumor burden and were comparable to tumor-naïve ctDNA liquid biopsy, especially as a single-marker test. These findings indicate that EDSA-BC has potential as a scalable and cost-efficient assay to aid in breast cancer management. Prospective studies are needed to clarify sampling tempo, clinical utility and benefit.

## Simple Summary

There is high interest in minimally invasive methods to track treatment response and detect recurrence in breast cancer patients. We previously discovered that novel protein biomarker BF-09 was measurable in serum and had high sensitivity and specificity across a broad range of breast cancer stages and subtypes. In this proof-of-concept retrospective longitudinal study, we wanted to study the serial dynamics of BF-09 in neoadjuvant, adjuvant and metastatic treatment follow-up to see if the marker had clinical validity in reflecting treatment response and recurrence. We observed that the biomarker had 83% accuracy in detecting cancer progression/remission and a median lead time of 9.4 months in detecting post-adjuvant relapse. The BF-09 immunoassay should be further studied as a scalable and cost-efficient method to aid clinicians in improving patient survival benefit.

## 1. Introduction

Breast cancer (BC) is the second-most diagnosed cancer in US women, with over 4 million BC survivors [1,2,3,4,5,6]. Current standard of care (SOC) for monitoring neoadjuvant, adjuvant or metastatic breast cancer therapy (NAT, AT, MBCT) efficacy is limited to advanced imaging (i.e., MRI, PET or CT), pathological staging at surgery and mammography at post-AT follow-ups [7,8,9,10,11]. Only 20–30% of BC patients achieve pathological complete response after NAT, and there are no recommended non-imaging methods for post-AT surveillance due to lack of proven survival benefit [10,12,13,14,15]. Therapy cycles typically run for 3–6 months or more and access to advanced imaging may be limited, meaning that patients risk harsh side effects for minimal benefit [16,17]. Due to a lack of active surveillance, distant recurrences are often not found until they become symptomatic due to imaging in the post-NAT/AT period being localized to the breast and chest area [8,18]. Increasing imaging frequency is impractical due to cost and radiation exposure [13].

Recommended alternatives for monitoring are limited. CA 15-3/CA-27/29 serum tests only have effective sensitivity in metastatic patients [19]. Histopathological marker and tissue multigene tests (i.e., Oncotype, MammaPrint) are one-time prognostic tests for determining treatment eligibility and stratifying recurrence risk, not for measuring therapy response [20,21,22,23,24]. Various versions of circulating tumor DNA (ctDNA) liquid biopsies are being heavily studied due to ease of sample draw and potentially wider clinical utility, although challenges remain regarding high cost, turnaround time and low titers [21,25,26,27,28,29]. Tumor-informed versions require a tumor sample to be sequenced for personalized mutation profiles while tumor-naïve tests only require blood to detect mutations and epigenetic markers.

An ideal test would be simple to perform, cost-effective, standardized and scalable. We previously described the discovery of a novel protein biomarker BF-09 for the early detection of breast cancer in serum [30], and a developed blood test, the Early Detection Serum-based Assay for Breast Cancer (EDSA-BC), showed a high sensitivity and specificity for differentiating BC from non-BC samples in single time points [31]. In this pilot proof-of-concept study, we wanted to explore de novo how the assay would behave longitudinally in detecting BC recurrence and relative tumor burden during different phases of treatment. The assay was used to analyze 379 retrospective longitudinal serum samples from 85 patients and determine its potential for further studies of clinical utility.

## 2. Materials and Methods

### 2.1. Patient Samples

All samples and patient clinical information were collected retrospectively under protocols and patient informed consent in place at the original institutions. Samples were handled and packaged by the provider’s institution, transported according to US and EU regulations as applicable and provided to Milagen in a de-identified manner. Samples were not obtained through any direct interaction or intervention and were provided with no identifiable private data or information (de-identified) by the provider’s institution and therefore did not qualify as Human Subjects Research under Title 45 CFR Part 46.102(e)(1)-(6) [32]. Samples were frozen on dry ice and immediately stored at −80 °C until use. Immediately prior to the assay, samples were aliquoted on ice to avoid multiple freeze–thaw cycles, and the original tube was maintained at −80 °C.

For the study, (*N* = 379) serum samples serially collected from 85 female (sex at birth) patients at various points of neoadjuvant, adjuvant therapy and metastatic breast cancer treatment (3–9 time points per patient) were retrospectively provided by the biobank at the IRCSS San Raffaele Pisana (Rome, Italy). Samples were collected from partner hospitals from 2007 to 2017. Clinicopathological features, including age, stage, grade, molecular subtype, receptor status and treatment type, of follow-up samples are provided in Table S1 and Supplementary Data S1. Tumors were classified into four subtypes as described by Orrantia-Borunda et al. [33]. Breast cancer subtypes were defined as follows: Luminal A (estrogen receptor (ER)-positive and progesterone receptor (PR)-positive and HER2-negative), luminal B (ER-positive and/or PR-positive, and/or HER2-positive), HER2 positive (HER2-positive and ER/PR-negative), triple negative breast cancer (ER/PR/HER2-negative). The cohort comprised four main subtypes: 31% luminal-A subtype (LLA), 48% luminal-B subtype (LLB), 18.8% triple negative (TN) and 3.5% HER2-positive, hormone-receptor-negative subtype (HER2+) (Table S1). Additionally, 67% of patients were diagnosed with stage I–III breast cancer while 33% had metastatic breast cancer. Treatments received by patients were neoadjuvant therapy (NAT, 16.5%), adjuvant therapy (AT, 18.8%), metastatic breast cancer therapy (MBCT, 35.3%) and combination therapy (AT ± NAT, MBCT, 29.4%). Tumor response to treatment (complete response, CR; partial response, PR; progressive disease, PD) was classified using Response Evaluation Criteria in Solid Tumors (RECIST) [34]. Patient’s status at follow-up was categorized as no evidence of disease (NED), alive with disease (AWD) or metastatic relapse (MET) by the attending physician based on clinical information.

Nine patients did not have enough clinical information and were excluded from the analysis. Four other patients were excluded due to lack of long-term follow-up data to interpret EDSA-BC performance but were discussed separately (Supplementary Materials). This left 313 samples from 72 patients available for primary analysis. Clinicopathological features are provided in Table 1. All 85 patients and the cohort of 72 patients used in the analysis had similar median age, molecular subtypes, tumor stage, grade compositions and proportion of treatment groups (Table S1 and Table 1). Median follow-up was 33.4 months (range 0–203 months). CA 15-3 levels measured at hospital visit (ADVIA Centaur assay; Siemens Healthineers AG, Munich, Germany) were provided for 58 patients.

### 2.2. ELISA Assay

Milagen EDSA-BC is an immunoassay based on a sandwich ELISA using a capture and a biotinylated detection monoclonal antibody (mAbs) matching pair against the BF-09 serum biomarker, and protocol details were described previously [30]. The assay uses serum-free cell culture medium (SFCM) from the MCF7 breast cancer cell line (Cat# HTB-22; RRID: CVCL_0031; ATCC, Manassas, VA, USA) as a surrogate calibrator antigen source and was optimized for BF-09 detection in human serum [35,36].

Performance characteristics of the assay have been determined: intra- and inter-assay reproducibility <15%CV (intra-assay: 5.4–6.5; inter-assay: 8.1–11.4) were within acceptable limits (Supplementary Material, Tables S2 and S3). Limit of detection (analytical sensitivity) and functional sensitivity were also previously described [30].

BF-09 concentration (μg equivalent/mL) in the serum of patients and controls (dilution 1:8) was determined using the EDSA-BC ELISA assay and was tested blindly with the investigator not knowing which samples belonged to which patient or stage of follow-up. Results were then interpolated from a calibration curve using MCF7 SFCM serially diluted using a 1:8 dilution of a normal human serum pool. A standard curve was generated using the 4-parameter logistic method (R^2^ = 0.992).

### 2.3. Statistical Analysis

EDSA-BC positivity or negativity was evaluated using two different classification criteria: static reference cutoffs and significant changes between serial samples (i.e., percentage change value). Static cutoff values were based on the 90th and 95th percentile of non-cancer patients analyzed in a previous early-detection study (57 and 86 µgE/mL, Supplemental Data S2) [31]. Another cutoff was calculated specifically for the present cohort using the 90th percentile of available baseline points at complete remission at surgery, samples within 1 year of AT start and points of long-term (3–6 years) follow-up with NED (151.2 µgE/mL; Supplemental Data S3). Points from partial remission at surgery or during MBCT were excluded to minimize the effect of residual disease in skewing biomarker levels. Samples with associated clinical status were analyzed to see if biomarker levels were above or below cutoff during disease progression or remission, respectively.

For serial dynamics, a change value of 30% was used to determine if a significant change beyond assay measurement variability occurred. This was based on 2.5× the assay’s total %CV of 10.7% (Supplementary Method). Percent change was calculated between on-treatment and post-treatment pre-surgery points (NAT phase) between samples within 1 year of AT start (median 3.6 months, range 0–15.4 months, except for 8 points within 3 years of AT start, median 36.7 months, 33.4–49.8 months) and either relapse (median 36.8 months/3.1 years, 3.5–56.6 months) or long-term post-AT surveillance (median 76.8 months/6.4 years, 55.8–109.8 months, with two samples at 30 and 43.6 months) and between point of diagnosis and first relapse (MBCT phase). For the CA 15-3 assay (ADVIA Centaur, Siemens) a significant increase was defined as >21% and a cutoff value of 32.4 U/mL to indicate elevated status was used based on the manufacturer’s published insert sheet.

Sensitivity was calculated as the percentage of patients with disease progression (PD, MET, secondary cancer) who were positive by EDSA-BC. Specificity was calculated as the percentage of patients who did not experience BC progression (NED, PR, CR) and tested negative. Positive predictive value (PPV) was calculated as [True positive/(True + False positive)] × 100. Negative predictive value (NPV) was calculated as [True negative/(True + False negative)] × 100. Accuracy (overall probability that a patient is correctly classified) is calculated as [(Sensitivity × Prevalence) + (Specificity × (1-Prevalence))]. Accuracy, PPV and NPV were calculated using MedCalc version 23.1.3 (RRID:SCR_015044; MedCalc Software Ltd, Ostend, Belgium) with a BC recurrence prevalence rate of 16.6% [37]. For comparison to liquid biopsy studies, LLA and LLB samples with ER ± PR+ were reconfigured as hormone receptor positive (HR+). Differences between median BF-09 levels were determined by a 2-sided Wilcoxon rank sum test.

Lead time was calculated as the time from first BF-09 serum rise to clinical recurrence or neoadjuvant response for patients with available samples. Ninety-five percent confidence intervals (CIs) were calculated using MedCalc version 23.1.3 and are reported for groups with sufficient sample numbers.

## 3. Results

### 3.1. Static Cutoff Analysis

Summary of analysis using static cutoffs is shown in Supplementary Table S4. The 57 µgE/mL cutoff had 80–81.3% SE in detecting post-NAT/AT BC progression at a low 14.3–21.1% SP. Using the 86 µgE/mL improved SP slightly at cost to SE. Both cutoffs had low SE for detecting PD/MET during MBCT. Neither was sufficient to show clinical validity in distinguishing BC progression from non-progression.

A cohort-specific cutoff of 151.2 µgE/mL was also calculated because the study population were BC survivors (instead of non-cancer) and because of the availability of longitudinal instead of single-point samples (Supplemental Data S3). Using this cutoff, correlation to clinical status for post-NAT/AT surveillance was low at 20.0–37.5% SE at 71.4–78.9% SP and 23.3% SE in MBCT (Table S4).

### 3.2. BF-09 Serial Dynamics and Relative Tumor Burden

Correlation to clinical status was also analyzed by serial dynamics, i.e., by increases/decreases in BF-09 above 30%, and are summarized by phase of treatment in Table 2. A rise in BF-09 serum levels had an overall per-sample accuracy of 83% (95% CI 74.2–89.9%) with 74% SE (95% CI: 62.0–84.2%) for BC progression and a fall had 85% SP (95% CI: 68.1–94.9%) for BC non-progression/remission across all phases of treatment, BC stages and histopathological subtypes. When only considering the most common BC subtype of HR+/HER2- (*n* = 48), per-sample detection accuracy was 86.7% (95% CI 76–93.8%) with 78% SE (95% CI: 61.5–89.2%) at 89% SP (95% CI: 69.9–97.6%). There was an insufficient number of other subtypes to calculate statistical significance. Performance in stage I–III follow-up (NAT + AT group) was 73% SE (95% CI: 52.2–88.4%) at 85% SP (95% CI: 68.1–94.9%). When including stage IV (all groups) SE changed slightly to 74% (95% CI: 62.0–84.2%) while SP remained unchanged (Table 2).

### 3.3. Monitoring Neoadjuvant Therapy

**Figure 1:**
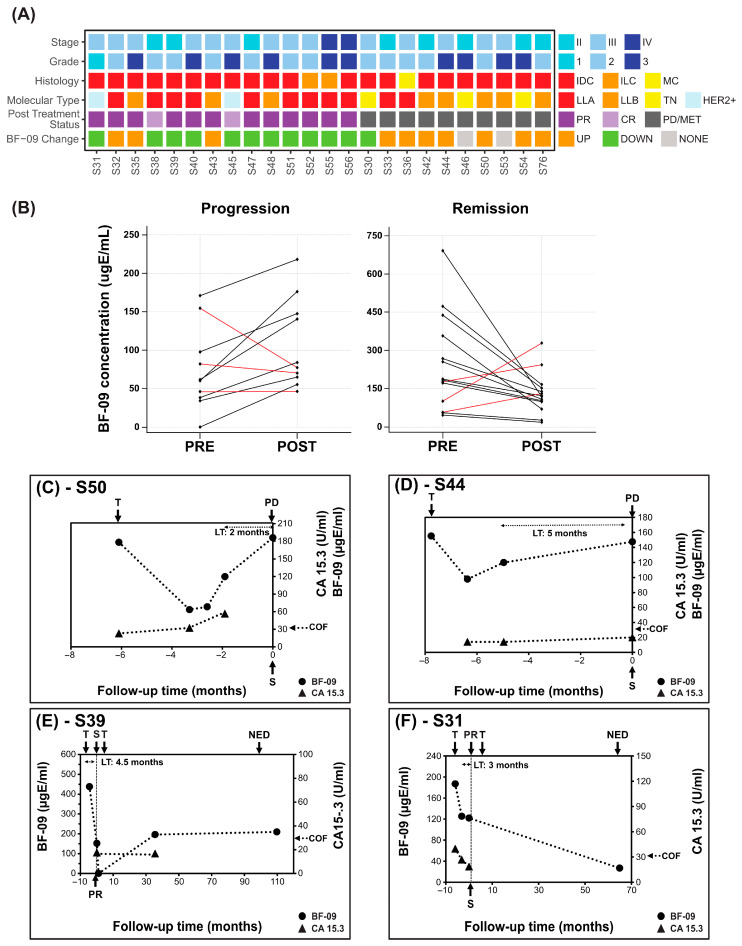
BF-09 and CA 15-3 dynamics in neoadjuvant treatment follow-up. (**A**) Summary of clinical characteristics of the cohort (stage, tumor grade, histological type, molecular subtype, patient post-treatment status (CR, PR, PD/MET)) and changes in BF-09 serum level from pre-surgical on-treatment to post-treatment (UP—increase; DOWN—decrease; NONE—unchanged); (**B**) Changes in pre-surgery on-treatment (PRE) and post-treatment, pre-surgery (POST) serum levels of BF-09 in individual patients grouped by patient treatment response (progression on left and remission on right). Black lines represent cases when BF-09 reflected clinical status and red lines represent cases when it did not. (**C**–**F**) Plots showing BF-09 levels (circle markers), as determined by EDSA-BC, and CA 15-3 (triangle markers) levels across time points in 4 representative BC cases. The *x*-axis tracks months leading up to (negative) and after (positive) primary surgery. Treatment start (T), surgery (S), complete (CR) or partial remission (PR) or no evidence of disease (NED) status of patient following treatment and CA 15-3 cutoff (COF) are indicated by arrows.

The study had 26 patients under NAT (16 stage III and 10 stage II) prior to surgery (Table S1). Twenty-two had enough samples and clinical information for analysis and data interpretation. Two stage IV BC patients who achieved partial remission while under treatment with PTX-BEV (one before surgery) were also included in this group for analysis as the intent of treatment was to shrink the tumor prior to possible surgery. Eighteen patients had CA 15-3 data available. The response rate was 50% (10 PR, 2 CR) with the rest having PD (*n* = 7 nodes, 1 local, 2 MET bone). On-treatment points ranged from 2–8 months before surgery. A grid chart showing key clinicopathologic characteristics and EDSA-BC test results is shown in Figure 1A.

A decrease >30% in BF-09 between on-treatment and post-treatment points was observed in 11 of 14 patients with tumor response after NAT (Figure 1B, 79% SP, 95% CI: 49.2–95.3%). Conversely, an increase in BF-09 was observed in 7 of 10 patients with BC progression (Figure 1B, 70% SE, 95% CI: 34.7–93.3%). The median of the difference between BF-09 level before and post treatment was statistically significant for cases of remission (*p* = 0.0419). The median of the difference could not achieve statistical significance for cases of progression among the small number of cases (*p* = 0.0840). There were not enough samples to analyze performance by subtype.

Median lead time determined for five patients with pre-surgical samples was 3.5 months (range 1.9–3.9 months).

Dynamics of BF-09 and CA 15-3 during treatment follow-up are shown for representative cases in Figure 1C–F. Additional treatment and clinicopathological details are shown in Table S5.

Patient #S50 (Figure 1C) reached an on-treatment low of 60.2 µgE/mL BF-09 which gradually rose to 176.2 µgE/mL (193%) at assessment of PD nodes. CA 15-3 increased from 22.9 to 56.6 U/mL (147%). Of note was that BF-09 seemed to capture initial treatment response before BC progression, but CA 15-3 did not.

Patient #S44 (Figure 1D) increased from 97.8 to 147.5 µgE/mL (50.8%) at PD nodes. CA 15-3 increased from 14.0 to 20.0 U/mL (42.9%) while remaining well below the cutoff.

Patient #S39 and #S31 (Figure 1E,F) had 65.3% and 34.8% decreases, respectively, in BF-09 levels at assessment of PR (decrease in tumor size ± nodal involvement, Supplemental Data S1). CA 15-3 similarly decreased 53.4% in #S31 (no data for #S39).

### 3.4. Post-Adjuvant Surveillance

**Figure 2:**
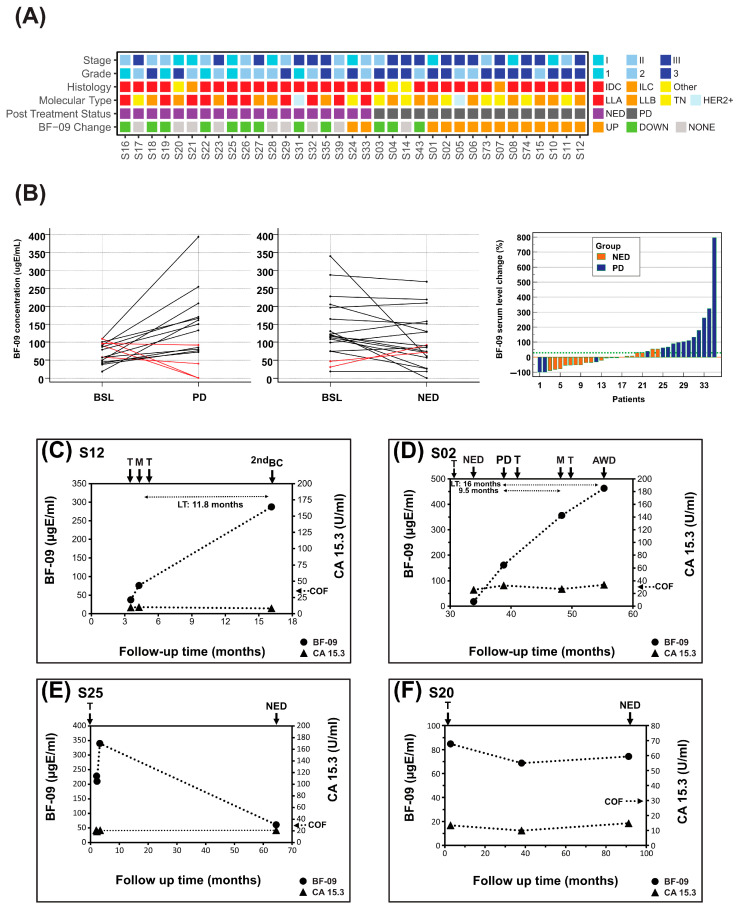
BF-09 and CA 15-3 dynamics during adjuvant treatment follow-up. (**A**) Summary of clinical characteristics of the cohort (stage, tumor grade, histological type, molecular subtype, patient post-treatment status (PD or NED)) and changes in serum level of BF-09 (UP—increase; DOWN—decrease; NONE—unchanged) associated with post-AT patient clinical status; (**B**) BF-09 serum level changes in individual patients between baseline (BSL, within 1 year of AT start) and relapse (left) or long-term NED (center). Waterfall plot showing the association of BF-09 dynamics with patient clinical status (PD—progression or NED—no evidence of disease) at long-term follow-up (right). Each bar represents an individual patient; (**C**–**F**) Plots showing BF-09 levels (circular markers), as determined by EDSA-BC, and CA 15-3 (triangular markers) levels across time points in 4 representative breast cancer (BC) cases. The *x*-axis tracks months since primary surgery. Treatment starts (T), metastatic relapses (M), progressive disease (PD), second breast cancer primary (2nd BC), alive with disease (AWD) or no evidence of disease (NED) status following treatment and CA 15-3 cutoff (COF) are indicated by arrows. Lead time (LT) is indicated for cases where samples are available.

The cohort had 40 patients (8 stage I, 14 stage II, 18 stage III) with samples from during and after AT. Thirty-five had enough samples and clinical information for analysis and data interpretation. Median follow-up was 66.3 months (5.3–148.9 months). The recurrence rate was 45.7% (*n* = 16; 6 nodes, 4 bone, 4 multisite, 2 new breast and colorectal), with the rest having NED at last follow-up (55.8–109.8 months). A grid chart of all 35 patients is shown in Figure 2A.

In addition, 75% (*n* = 12/16, 95% CI: 47.6–92.7%) of patients with BC progression had levels of BF-09 that increased by more than 30% at relapse compared to BSL while 89% (*n* = 17/19, 95% CI: 66.9–98.7%) with long-term NED had either a decrease or no change (Figure 2A,B). SEs by stage, subtype and relapse location are shown in Table S6 (statistical significance not possible due to sample size).

BF-09 level changes correlated well with clinical response to AT treatment (Figure 2B, right) as determined by SOC (imaging). Rate of long-term NED was 81% (*n* = 17/21) for patients with decreased or unchanged BF-09 but only 14.3% (*n* = 2/14) for patients with an increase in BF-09. Using a Wilcoxon paired sample test, median BF-09 levels were found to be significantly higher at PD/MET points compared to baseline (*p* = 0.0290, Figure S1A) and borderline lower/stable at long-term NED follow-up (*p* = 0.0582, Figure S1B).

Dynamics of BF-09 and CA 15-3 during AT and follow-up for representative cases are shown in Figure 2C–F. Treatment data and clinical information are shown in Table S7. 

Patient #S12 (Figure 2C) had BF-09 level of 37.4 µgE/mL at start of AT which increased 104% to 76.1 µgE/mL at diagnosis of MET lymph nodes. Biomarker levels rose another 277.5% by diagnosis of second breast cancer, despite transitioning the patient to hormonal and radiotherapy. By contrast, CA 15-3 levels remained steady within a normal range during the same period.

Patient #S02 (Figure 2D) had a biomarker level of 18 µgE/mL at start of AT which rose 798% to 161.7 µgE/mL by clinical assessment of PD nodes. After a regimen change, BF-09 increased 120% further to 356 µgE/mL at diagnosis of MET lung/liver and rose again by 30% to 463 µgE/mL concurrent with remaining disease at last follow-up of AWD. CA 15-3 levels were inconsistent and borderline, rising 26.4% at PD nodes but not at MET lung/liver.

Patient #S25 (Figure 2E) had BF-09 serum level of 219 ugE/mL at start of AT which rose 55% to 340 µgE/mL after 1 month. This later decreased by 82% to 61 ugE/mL at clinical assessment of NED. CA 15-3 remained stable, below cutoff during the same period.

Patient #S20 (Figure 2F) had BF-09 level of 85 µgE/mL at start of AT, which did not change above 30% at either the 38.1 or 91.2 long-term follow-ups. This was associated with a clinical status of NED. CA 15-3 levels remained below cutoff and did not increase during the same period.

### 3.5. Monitoring Metastatic Breast Cancer Therapy

**Figure 3:**
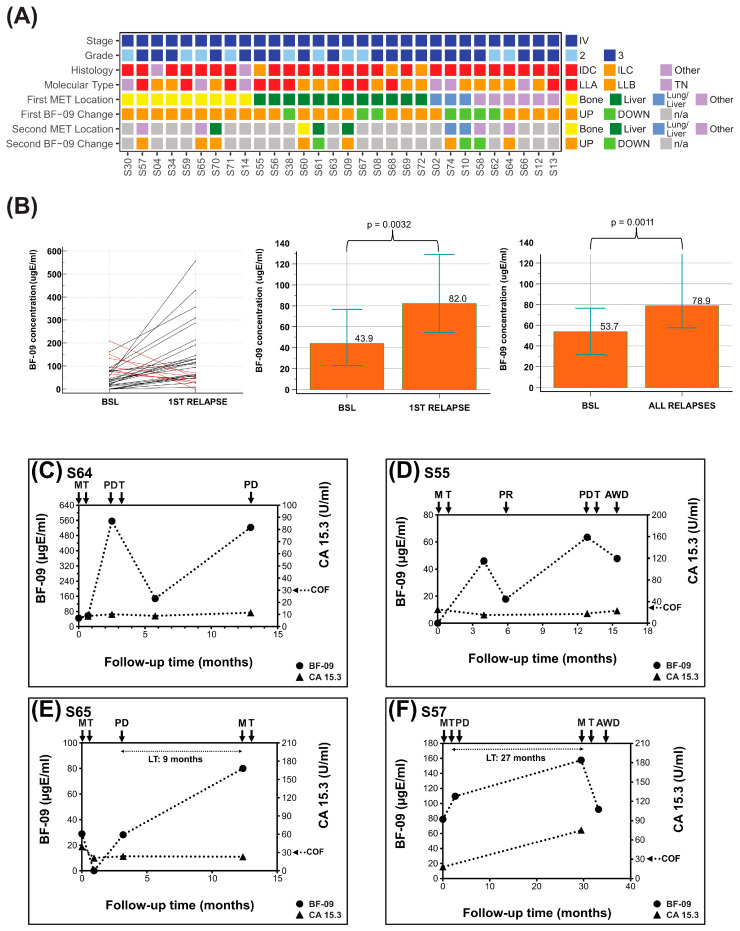
BF-09 and CA 15-3 dynamics during metastatic breast cancer treatment follow-up. (**A**) Summary of clinical characteristics of the cohort (stage, tumor grade, histological type, molecular subtype, patient post-treatment metastasis location (first ± second MET)) and on-treatment changes in serum level of BF-09 (UP—increase; DOWN—decrease) associated with patient clinical status; (**B**) BF-09 serum levels changes in individual patients between baseline (BSL) and first relapse. Black lines represent cases when BF-09 reflected clinical status and red lines represent cases when it did not. Column plots showing median BF-09 serum level at baseline (BSL, diagnosis of metastasis) and points nearest first relapse (left, *p* = 0.0032) and points nearest all relapses (right, *p* = 0.0011) by Wilcoxon test of paired samples. Overlaid error bars represent 95% confidence interval. (**C**–**F**) Plots showing BF-09 levels (circular markers), as determined by EDSA-BC, and CA 15-3 (triangular markers) levels across time points in 4 representative breast cancer (BC) cases. The *x*-axis tracks months since metastatic diagnosis. Treatment start (T), metastatic relapse (M), partial remission (PR), progressive disease (PD) or alive with disease (AWD) status following treatment and CA 15-3 cutoff (COF) are indicated by arrows. Lead time (LT) is indicated for cases where samples are available.

Twenty-nine stage IV patients received MBCT, with twenty having adequate samples and clinical information for analysis. In addition, 10 were transitioned to MBCT after distant recurrence following NAT/AT, for a total of 30 patients. Fourteen patients had recorded levels of CA 15-3 at the start of therapy and at some follow-up points. A grid chart of all 30 patients and important characteristics is shown in Figure 3A.

For the first relapses after MET diagnosis (i.e., per-patient sensitivity, *n* = 30 relapses from 30 patients), 76.7% (*n* = 23/30, 95% CI: 57.7–90.1%) were correlated with more than a 30% increase in BF-09 (Table 2). As some patients had multiple relapses (i.e., per-sample sensitivity, n = 40 relapses from 30 patients), SE was 75% overall (*n* = 30/40, 95% CI: 58.8–87.3%, Table 2). Median biomarker levels at first relapse and all relapses were significantly higher than at diagnosis (*p* = 0.0032, *p* = 0.0011, Figure 3B). Sensitivities by major MET sites were 91% for any with bone involvement (*n* = 10/11), 73.3% for liver (*n* = 11/15) and 71.4% for lung (*n* = 5/7; Table 3). There were not enough samples to analyze performance by subtype.

Similar SE by site was observed when combining all recurrences (*n* = 56) detected by BF-09 while under MBCT or AT (*n*= 39) with the addition of 83.3% SE for nodes (*n* = 5/6) and 100% for locoregional relapse (*n* = 4/4, Table S8).

In post-AT and MBCT follow-up, 10 patients had pre-relapse samples. The median lead time was 9.4 months (range 0.8–27 months).

The graphs of four representative MBCT patients are shown in Figure 3C–F. Group data are shown in Table S9.

Patient #S64 (Figure 3C) had BF-09 level of 45 µgE/mL at start of first-line treatment which increased 1141% to 556 µgE/mL at clinical diagnosis of PD lung. Serum marker level decreased 73% to 148 µgE/mL after start of second-line treatment but increased again by 253% to 523 µgE/mL at further PD lung. CA 15-3 serum levels during this same period remained flat and below cutoff except for a 29.2% increase at second PD lung while staying well below cutoff.

Patient #S55 (Figure 3D) had BF-09 level of 46 µgE/mL after start of first-line treatment which decreased 61% to 18 µgE/mL at clinical assessment of PR. Biomarker level then rose 253% to 63 µgE/mL at PD liver before decreasing to 48 µgE/mL after starting second-line therapy and at last follow-up of AWD. CA 15-3 remained below cutoff the entire period and did not increase at PD liver but only at the last follow-up point.

Patient #S65 (Figure 3E) had BF-09 level of 28.8 µgE/mL at start of first-line treatment which initially decreased to below the assay detection limit before increasing back to 28.1 at PD bone and another 184% to 80.0 µgE/mL at MET lung. CA 15-3 levels initially decreased after first-line treatment but did not increase at either progression event.

Patient #S57 (Figure 3F) had BF-09 level of 78.81 µgE/mL at start of first-line therapy which rose 38.9% at PD bone and another 43.9% at MET lung. After start of second-line treatment, biomarker level decreased 41.6% at last follow-up of AWD. CA 15-3 rose 317% at MET lung (no data for PD bone).

### 3.6. Therapy Type Association

Sensitivities of EDSA-BC by AT/MBCT regimen types are shown in Table S10. Notably, an increase in BF-09 had 89.5% SE for relapse while on combinations of taxane and targeted therapies (*n* = 17/19) and 88.9% SE for combination chemotherapies excluding taxane (*n* = 8/9). SE was lowest when on taxane only (*n* = 1/6).

## 4. Discussion

EDSA-BC is an immunoassay using monoclonal antibodies against protein target SAGA-associated factor 29 (SGF29), as previously described [30,38,39,40,41]. Our research indicates SGF29 is measurable in serum when secreted as part of a malignant cellular process or when tumor cells die and release their contents into the bloodstream [42,43].

### 4.1. Serial Dynamics

Serial dynamics have been applied previously to biomarkers used in cancer management such as CA 15-3 and lately to ctDNA where changes over time were more informative and predictive of disease status [44,45,46,47,48,49,50,51,52]. Approaches that rely on two or more time points to describe changes in biomarker values may also better characterize the complex kinetics of tumor markers during treatment [53]. We applied the same analytic approach to EDSA-BC here.

We showed previously that BF-09 serum levels were higher in the presence of BC and progressive disease, so elevated baselines in some patients (Figure 2B, center) could have been due to residual disease not detected by clinical imaging at the time [30,54,55,56]. Those whose high baselines remained unchanged even at long-term NED (Figure 2B, center) could also be explained by physiological reactions to therapy [57,58,59,60].

### 4.2. BF-09 Levels and Relative Tumor Burden

Using clinical status as proxies for tumor burden, changes in BF-09 serum levels above 30% reflected tumor changes across NAT, AT and MBCT with 74% SE (PD, METS) and 85% SP (PR, CR, NED) for all BC pathologic subtypes and 78% SE and 89% SP for HR+/HER2- only (Table 2). This showed a strong degree of correlation between BF-09 and relative tumor burden, indicating that EDSA-BC could be the basis of an alternate way to gauge patient response in between imaging rounds. NPV was high at 94.3–95.2%, which would be of value in reassuring clinicians that a decrease or long-term post-AT stabilization of BF-09 levels is predictive of positive outcomes for the patient (remission/NED).

Single-site sensitivity for PD/MET was highest in bone (90.9%), liver (73.3%) and lungs (71.4), which represent up to 78% of single-site BC MET (Table S8) [61,62]. Interestingly, multisite MET SE was lower (57–60%) albeit the sample size was small. This difference in biomarker behavior may reinforce the notion that multisite metastasis is a distinctly challenging group [63,64].

### 4.3. Post-Adjuvant Surveillance and MRD

An increase in BF-09 after AT treatment was indicative of minimal residual disease (MRD) and disease progression with 75% SE at 89.5% SP (Table 2). This was comparable, especially considering EDSA-BC is a single-marker tumor-naïve test, to more complex ctDNA liquid biopsies being studied [26]. The Signatera MRD, a tumor-informed assay, was reported to have 88.2% SE at 95.1% SP overall, and Guardant Reveal, a tumor-naïve assay, reported 28.9–79% SE at 97.7–100% SP overall [50,65,66,67]. BF-09 showed strong performance as a single biomarker for MRD. This was probably due to the multistep screening that was geared to identify a biomarker applicable to different subgroups of BC [30].

In HR+/HER2- cancers, EDSA-BC SE was 100% and compared well to 81.8% and 80-100% for Signatera (*n* = 18/22) and Guardant Reveal (*n* = 8/10, 8/8), respectively (Table S6) [50,65,66]. In the TNBC and HER2+ subtypes, assay sensitivities of 71.4% and 100% were comparable to 100% in both by Signatera (*n* = 7/7, *n* = 2/2) and 25–75% for TNBC by Guardant Reveal (*n* = 2/8, *n* = 3/4) [50,65,66].

Based on the small sample number, EDSA-BC SE for stage I relapse was 100%, which was on par with a similarly sized cohort reported by Signatera (100%, *n* = 2/2, Table S6) [50]. Lower SE in stages II and III was discordant to our previous study in a larger dataset (*N* = 433) [31]. A larger longitudinal cohort with more purposefully spaced sampling will be needed to confirm.

EDSA-BC SE for secondary cancers (breast, colorectal) was 100% (Table S6). If confirmed in a larger cohort, this would be significant as tumor-informed assays may not detect these cancers due to constant genetic changes and extreme variability [26,47,68,69].

EDSA-BC’s median lead time of 9.4 months in predicting relapse prior to clinical diagnosis compared well to Signatera’s 10.5 months (0–38 months) and Guardant’s 5.1–10.3 months (0–38 months) [50,65,66,67].

EDSA-BC has shown its potential for early detection with high SE and SP. We hypothesize that it would be worth conducting additional studies to possibly demonstrate that the detection of locoregional or secondary relapses can improve survival [70].

### 4.4. Monitoring Neoadjuvant Treatment Response

In the NAT phase, BF-09 levels significantly decreased in 78.6% in patients with PR/CR at surgery, showing promising predictive ability for treatment benefit (Table 2). In the two patients who achieved CR, SE was 100% (*n* = 2/2, Figure 1A). It is possible that some patients who saw an increase in BF-09 despite achieving PR may have been showing micrometastasis outside the breast, but samples and information were not available to confirm.

Studies using Signatera during NAT reported a 94.7% association of ctDNA clearance and decrease in tumor volume in TNBC and a 100% association of ctDNA clearance and CR in HR+/HER2-, TNBC and HER2+ patients [69,71]. A focus of liquid biopsy studies was the correlation between detectable ctDNA after NAT and shorter overall and progression-free survival [26,44,65,69,72].

Imaging during NAT is not standardized and months may pass without the clinician knowing if the therapy is actually working [8,73]. Critical windows of opportunity may be missed, as shrinking tumors increase chances of curative success. In patient #S50 (Figure 1C), BF-09 level was at its minimum more than 3 months prior to surgery before rising again prior to confirmation of PD at surgery, meaning that, instead of scheduling surgery at maximum treatment efficacy, the tumor had three additional months to grow.

### 4.5. Monitoring Metastatic Treatment Response

During MBCT, BF-09 rose in 76.7% of patients at first relapse/PD following diagnosis and in 75% of all progression events (Table 2). EDSA-BC showed high SE in detecting progression to the bone (Table 3), a distal recurrence site that has presented difficulties to ctDNA assays [48,49,50,66,68].

When treating metastatic BC, it is important to know not only if therapy works but also when it stops working so the treatment plan can be reconsidered to spare patients unnecessary treatment toxicities and preserve the quality of life. For example, in patient #S02 (Figure 2D, Table S7), the second-line treatment was the same as that used in the AT phase (i.e., Taxotere, TC). BF-09’s continual increase post MET signaled tumor adaptation and treatment resistance, as confirmed by no indications of remission at last follow-up.

In this study, EDSA-BC had high SE in detecting BC progression (i.e., treatment resistance) across most types of treatment involving different mixes of chemotherapies and targeted therapies, indicating possible wide AT and MBCT population applicability (Table S10).

### 4.6. Other Observations

In some patients, such as #S25 and #S55 (Figure 2E and Figure 3D), BF-09 level increased in early therapy cycles before decreasing at PR or NED. This phenomenon has also been seen in other protein markers like CA 15-3 and may be a result of either delayed treatment response or dying tumor cells releasing their contents into circulation [8,74].

CA 15-3 results were consistent with published findings [8,19]. In non- or pre-metastatic patients, the biomarker level often remained below the manufacturer’s 32.4 U/mL cutoff limit despite changes in clinical status. In metastatic patients, the marker was inconsistent in rising above the cutoff. In patients #S02, #S12, #S55 and #S64 (Figure 2C,D and Figure 3C,D) CA 15-3 levels remained below cutoff, essentially flat, and non-informative of treatment effect. It did increase above cutoff at MET in patient #S57 (Figure 3F).

### 4.7. Immunoassay Format Advantages over ctDNA Liquid Biopsy

Because EDSA-BC is a simple ELISA test detecting a protein in serum, it can potentially be integrated into existing clinical blood test workflows and tracked through medical records systems in place. Pending future validation, possible utilities include gauging whether NAT patients can have earlier surgery. In post-AT surveillance, it could be a warning to increase follow-up intensity and trigger advanced imaging. During MBCT, it could alert clinicians to when treatment becomes ineffective and possibly spare patients from unnecessarily prolonged toxicity exposure. The antibody-based nature of the assay makes it possible for the assay to be automated and scaled like many other protein-based ELISAs (i.e., CEA, CA 15-3, etc.), which should present significant cost savings relative to genetic testing. Turnaround time for a tumor-naïve immunoassay would also be significantly shorter than genetic tests which could take half a month or more [26,66]. EDSA-BC is currently planned to require no more than 5 mL of blood while ctDNA marker tests require a minimum of 20 mL blood volume as a hedge against low titers of genetic markers, especially with locoregional recurrences [25,26,47,75]. The short half-life of ctDNA in blood samples may present specific technical and logistic issues, and dependency on detecting mutations makes liquid biopsies not applicable to wild-type tumors [25,68]. Additionally, EDSA-BC is tissue-naïve, which is important as up to 41% of patients may not have sufficient tumor volume for analysis after NAT and mutations may change as cancer evolves [26,66,69].

### 4.8. Strength and Limitations

The main limitations were the small subgroup sizes and lack of repeated blood sampling before and after treatment points which prevented a more precise study of biomarker dynamics, lead times and correlation of BF-09 dynamics with overall survival or progression-free survival (i.e., clinical benefit). Specific indications and performance differences between BC subtypes could not be determined. In addition, some EDSA-BC false-positive discordance may have been due to non-imaging of distant sites based on the current recommendations of not getting extensive imaging for surveillance.

Prospective studies will be needed to recruit a larger number of patients from different subgroups, take samples at pre-defined intervals around treatment and calculate survival benefit in order to validate the clinical utility and benefit of EDSA-BC. A larger independent validation set is also needed to confirm results and determine if the biomarker behaves differently between subtypes.

One strength from using banked retrospective samples was access to longer post-AT follow-up (median = 66.3 months, range 5.3–148.9) which allowed for long-term NED assessment.

## 5. Conclusions

In summary, this preliminary feasibility study showed strong clinical validity that BF-09 correlated with relative tumor burden in NAT, MBCT and post-AT surveillance and can potentially serve as a test for treatment guidance. While the liquid biopsy space has focused on the same possible utilities by measuring ctDNA, this is the first time that a serum protein-based immunoassay has demonstrated such capability and potential. Further testing in prospective trials is needed to confirm clinical utility and benefit.

The EDSA-BC test was developed to differentiate between normal and various stage/subtypes of BC and as such showed a broad applicability to a variety of treatments and patient groups regardless of genetic variability and recurrent mutations. As a blood-based immunoassay, it is minimally invasive, cost effective, reproducible and scalable, which would allow its use in standard clinical settings as frequently as needed compared to medical imaging.

## 6. Patents

Results from this study have been submitted by Dr. Moncef Jendoubi to the US Patent Office and are patent pending to Milagen.

## Figures and Tables

**Table 1:** Clinicopathological characteristics of patients at baseline.

Characteristics	Year	Range
**Median age at diagnosis**	50	28–75
**Characteristics**	**N**	**(%)**
**Estrogen receptor status**		
Positive	53	73.2
Negative	19	26.8
**Progesterone status**		
Positive	49	68
Negative	23	32
**HER2 status**		
Positive	10	13.9
Negative	62	86.1
**Molecular type**		
Luminal A	25	35.7
Luminal B	30	41.7
Triple negative	14	19.4
HER2-positive	3	4.1
**Histology at diagnosis**		
Invasive ductal carcinoma	62	86.1
Invasive lobular carcinoma	7	9.7
Other	3	4.1
**Tumor stage**		
I	8	11.1
II	18	25
III	26	36.1
IV	20	27.8
**Grade**		
1	5	6.9
2	28	38.9
3	36	50.0
Unknown	3	4.2
**Treatment**		
NAT	13	17.8
AT	16	21.9
MBCT	21	28.8
NAT+ AT	6	8.2
NAT ± AT and MBCT	5	6.8
AT + MBCT	12	16.4

Clinicopathological characteristics of patients at baseline. Set of 72 patients for analysis of EDSA-BC performance. Nine patients were excluded from the analysis due to the lack of sufficient clinical information. Four other patients were excluded from the analysis due to lack of long-term follow up data to interpret EDSA-BC performance but are discussed separately (Supplementary Material). Abbreviations: Neoadjuvant therapy (NAT), Adjuvant therapy (AT), metastatic breast cancer therapy (MBCT), number of patients (N).

**Table 2:** EDSA-BC assay performance as measured by changes >30% in detecting progressive disease during neoadjuvant, adjuvant and metastatic treatment follow-up.

Application	Patients (N)	Follow-Up Event (N)	PD Events	No PD Events	SE (%)/ 95% CI	SP (%)/ 95% CI	PPV/ 95% CI	NPV/ 95% CI
NAT	24	24	10	14	7/10 (70%)/ 34.7–93.3%	11/14 (79%)/ 49.2–95.3%	39.4%/ 18.1–65.7%	92.9%/ 83.1–97.2%
AT	35	35	16	19	12/16 (75%)/ 47.6–92.7%	17/19 (89%)/ 66.9–98.7%	58.7%/ 27.1–84.4%	94.7/ 88.4–97.7%
Early BC Follow-up (NAT + AT) *	53	59	26	33	19/26 (73%)/ 52.2–88.4%	28/33 (85%)/ (68.1–94.9%)	49.0%/ 29.3–67.0%	94.1%/ 89.2–96.8%
MBCT	30	30	30	0	23/30 (77%)/ 57.7–90.1%	NA	NA	NA
MBCT *	30	40	40	0	30/40 (75%)/ 58.8–87.3%	NA	NA	NA
All Groups *	72	99	66	33	49/66 (74%)/ 62.0–84.2%	28/33 (85%)/ (68.1–94.9%)	49.4%/ (30.0–68.9%)	94.3%/ (91.5–96.2%)
All Groups—HR+/HER2-SUBTYPES *	48	66	40	26	31/40 (78%)/ 61.5–89.2%	23/26 (89%)/ 69.9–97.6%	57.2%/ 31.3–79.7%	95.2%/ 91.6–97.3%

EDSA-BC assay performance as measured by changes >30% in detecting progressive disease during neoadjuvant, adjuvant and metastatic treatment follow-up. EDSA-BC performance in 72 patients (99 disease status events) grouped by neoadjuvant, adjuvant and metastatic breast cancer therapy phases. Early BC Follow-up includes stages I–III while All Groups includes stages I–IV. Number (N) of patients and events, sensitivity (SE) for progressive disease/metastasis, specificity (SP) for remission/NED, positive predictive value (PPV) and negative predictive value (NPV) reported as fraction, % estimate and 95% confidence interval range as appropriate. Abbreviations: NAT—neoadjuvant, AT—adjuvant, MBCT—metastatic breast cancer treatment, HR—hormone receptor positive, N—number, PD—progressive disease, SE—sensitivity, SP—specificity, PPV—positive predictive value, NPV—negative predictive value, CI—confidence interval. *—per sample sensitivity (encompasses all relapses including patients who had multiple).

**Table 3:** Per-sample sensitivity of MBCT patients by relapse site.

Location of Recurrence	N	Detected (Ratio)	Detected (%)
Bone+/− Other Sites (Brain, Liver, Nodes, Lung)	11	10/11	91.0
Liver	15	11/15	73.3
Lung	7	5/7	71.4
Lung + Liver	4	2/4	50.0
Locoregional	2	2/2	100.0
Brain	1	0/1	0.0
Total	40	30/40	75.0

Per-sample sensitivity of MBCT patients by relapse site. Counts of how many relapses by site were observed in this cohort. Includes all metastatic time points from all MBCT patients (*n* = 40 relapses from 30 patients). Also shown are ratio and percentages of how many recurrences detected by increases in BF-09 serum level over total number of relapses per site.

## Data Availability

The original contributions presented in this study are included in the article/Supplementary Material. Further inquiries can be directed to the corresponding author. The assay used in this study is proprietary to Milagen.

## Supplementary Materials

The following supporting information can be downloaded at: https://www.mdpi.com/article/10.3390/cancers17244004/s1, Supplementary Materials—Supplementary Methods, Table S1: Clinicopathological characteristics of patients at baseline, Table S2: Assay precision and variability, Table S3: Sensitivity and Specificity at different values of BF-09 percent change, Table S4: Sensitivity and Specificity of EDSA-BC when using static cutoffs, Table S5: Summary of representative patient characteristics during NAT treatment follow-up, Table S6: Sensitivity of AT surveillance by stage, subtype and relapse location, Table S7: Summary of representative patient characteristics undergoing adjuvant therapy treatment follow up, Table S8: Per-sample sensitivity of AT and MBCT patients by relapse site, Table S9: Summary of representative patient characteristics undergoing metastatic breast cancer treatment follow-up, Table S10: Sensitivity of EDSA-BC in AT and MBCT by therapy regimen type, Figure S1: Comparison of median BF-09 serum levels after adjuvant treatment, Supplementary Text: Excluded patients’ analysis (pdf), Supplementary Data S1: Patient Clinical information and EDSA-BC test results, Data S2: BF-09 reference cutoff in non-cancer population based on 90th and 95th percentile, Data S3: BF-09 reference cutoff in BC survivors’ baselines based on 95th percentile, Data S4: EDSA-BC assay %CV calculation dataset (pdf).

## Abbreviations

The following abbreviations are used in this manuscript:

AT	Adjuvant therapy
AWD	Alive with disease
BC	Breast cancer
BSL	Baseline
CEA	Carcinoembryonic antigen
CFR	Code of Federal Regulation
CI	Confidence interval
CR	Complete response
CT	Computed tomography
ctDNA	Circulating tumor DNA
CV	Coefficient variation
EDSA-BC	Early Detection Serum-based Assay for Breast Cancer
ELISA	Enzyme-linked immunosorbent assay
ER	Estrogen receptor
EU	European Union
HER2	Human epidermal growth factor receptor 2
LLA	Luminal A
LLB	Luminal-B subtype
mAbs	Monoclonal antibody
MBCT	Metastatic breast cancer therapy
MET	Metastatic relapse
MRD	Minimal residual disease
MRI	Magnetic resonance imaging
NAT	Neoadjuvant therapy
NED	No evidence of disease
NPV	Negative predictive value
PD	Progressive disease
PET	Positron emission tomography
PPV	Positive predictive value
PR	Progesterone receptor
PR	Partial response
PTX-BEV	Paclitaxel–Taxotere
SE	Sensitivity
SFCM	Serum-free cell culture medium
SGF29	SAGA-associated factor 29
SOC	Standard of care
SP	Specificity
TC	Taxotere/Cyclophosphamide
TNBC	Triple negative breast cancer
US	United States
