# Supplementary material for: A Novel Protein Serum Biomarker Assay for Tracking (Neo)adjuvant and Metastatic Therapy Efficacy and Enabling the Timely Detection of Relapse in Breast Cancer
Source: Cancers (Basel). 2025 Dec 16;17(24):4004. doi: 10.3390/cancers17244004 (PMC12731775; doi:10.3390/cancers17244004)
Supplement: Supplementary file 1 [file cancers-17-04004-s001.zip › Supplementary Data S1.pdf]

SERUM CODE	AGE	Histology	G	ER (%)	PR (%)	HER2 /NEU	Molecular Type	Stage	TNM	Follow-up Days	Follow-up months	BF-09 µgE/ml	CA15.3 U/ml	STATUS	Treatment type	Regimen
10568	74	IDC	3	0	50	NEG	LLB	IA	pT1N0	0	0.0	57.7	36.9	NED		
13847										1118	37.3	< Curve				
24296										1601	53.4	< Curve	757.0	MET bone	I line	VNR + HERC
24373										1608	53.6	80.3		AWD		
5622	53	IDC	3	0	0	NEG	TN	IIIC	pT2N3	1016	33.9	18.0	25.8	NED	ADJ	TC
7085										1164	38.8	161.7	32.6	PD Nodes	I line	Capecitabine
11100										1450	48.3	356.2	27.0	MET Lung/Liver	II line	TC
13214										1658	55.3	463.0	33.6	AWD		
21408	48	IDC	3	0	0	NEG	TN	IIA	pT2N0	1208	40.3	159.3	19.3	NED	ADJ	Aromatase inhibitors
21645										1230	41.0	58.2	20.4			
24150										1421	47.4	40.0	30.2	MET Bone/Lung	I line	Myocet/cyclophosphamide
24400										1441	48.0	< Curve	35.3	AWD		
6209	61	Mucinous	3	30	30	POS	LLB	IIIB	T4N3	1494	49.8	90.5	33.5	NED		
6575										1528	50.9	< Curve	39.9	MET Nodes	I line	PTX-HERC
6899										1570	52.3	< Curve	31.4			
28258										4467	148.9	8.5		MET Bone		
2099	64	IDC	2	0	0	POS	HER2+	IIIA	pT1N2	91	3.0	86.4	8	NED	ADJ	FEC-TXT
2644										139	4.6	93.2	8.3			
7591										610	20.3	170.4		CRC pT4N1Mx, IIIB	Surgery	
10519										797	26.6	125.6			ADJ	FOLFOX
11352										860	28.7	138.9		NED		
14975	46	IDC	2	90	70	NEG	LLB	IIIB	pT2N2	0	0.0	109.1		NED	ADJ	AC-TXT
15169										21	0.7	163.9				
15738										63	2.1	225.6				
16190										105	3.5	393.9		MET Bone 1.8 months later.	I line	PTX-BEV (at MET Bone)
17247	72	ILC	3	0	0	NEG	TN	IIIC	pT2N3	40	1.3	176.3	32.1	NED	ADJ	AC/TXT
17995										125	4.2	91.4				
22122										947	31.6	100.8		MET Bone	I line	TXT-HERC
23901										1083	36.1	254.6		AWD		
6694	67	IDC	3	50	20	NEG	LLB	IA	pT1N0	99	3.3	79.0	18.9	NED	ADJ	FEC
6892										820	27.3	73.7				
17225										1042	34.7	74.6		MET Lung/bone		
17785										1121	37.4	150.1			I line	PTX
22341										1989	66.3	< Curve		MET Liver		
24443	58	IDC	2	60	0	NEG	LLA	IV	pT2N3	0	0.0	11.5			Surgery	
25577										106	3.5	48.4		MET Liver	I Line	PTX-BEV
25801										399	13.3	59.8		PD Liver		
6797	64	IDC	3	50	20	NEG	LLB	IA	pT1cpN0	134	4.5	57.1	18.0	NED	Adjuvant	FEC
17690										1135	37.8	132.8	41.0	MET lung/LN	First-line	Paclitaxel
42205										1717	57.2	69.6		MET Liver/Lung		
22244										2003	66.8	20.3	123	PD Lung/Liver/Bone	Second-line	Eribulina
15869	47	IDC	3	0	0	NEG	TN	IIB	pT2pN1	43	1.4	210.7	14.6	NED	Adjuvant	AC-Taxotere
16147										66	2.2	40.6	25.4			
87303										543	18.1	85.5		MET LN	First-line	Paclitaxel
20156										550	18.3	63.0	30.8	DOD		
219	46	IDC		60	50	NEG	LLB	IIIB	T2N2	106	3.5	37.4	9.5	NED	Adjuvant	FEC
417										128	4.3	76.1	10.1	MET LN		Hormone/Radiotherapy
4203										484	16.1	287.3	8.2	2nd Breast Cancer	Surgery	Hormone/Radiotherapy
4205										2902	96.7	66.8		3rd Breast Cancer	Surgery	
28188										3635	121.2	36.8		AWD		
6751	58	IDC	3	0	30	NEG	LLA	IV	n/a	0	0.0	18.3	25.4	NED	Adjuvant	FEC
7217										57	1.9	146.8				
8045										145	4.8	267.0				
9724										229	7.6	226.5	28.6			
46106										413	13.8	191.9	23.2	PD lung	First-line	Herceptin
17708										1009	33.6	134.7	27.9	DOD		Vinorelbine/Herceptin
17152	72	LC pleomorp	3	0	0	NEG	TN	IIIC	pT2pN3	54	1.8	282.1	34.1	NED	Adjuvant	AC-Taxotere
17326										76	2.5	132.3	29.9			AC-Taxotere
17900										139	4.6	96.2	26.3			AC-Taxotere
94104										775	25.8	91.8	32.3	MET Bone/LN		
22027										961	32.0	67.9	63.8	PD	First-line	Herceptin/Taxotere/Pertuzumab
22391										992	33.1	309.0				Herceptin/Taxotere/Pertuzumab
23806										1097	36.6	454.3	33.6			Herceptin/Taxotere/Pertuzumab
28594										2035	67.8	32.7		AWD		
13687	37	IDC	2	30	10	POS	LLB	IIIC	cT4cN3	72	2.4	221.7	18.6	NED	Adjuvant	Aromatase inhibitor/Tamoxifen
13865										100	3.3	243.4				Herceptin
14191										141	4.7	283.4	20.8			
14775										211	7.0	100.2				
14524										853	28.4	166.4	15.5	2nd Breast Cancer	Surgery	
64207										1064	35.5	97.6	9.0	PD LN		
24450										1527	50.9	48.4	44.6	AWD	First-line	Herceptin/Pertuzumab
7743	38	IDC	1	90	20	NEG	LLA	IIB	pT2N1	0	0.0	30.82	10.1	NED	ADJ	AC-TXT
8844										62	2.1	141	26.6			
11298										227	7.6	74.96	9.0			
13733										1479	49.3	180.5	24.6			
13883										2767	92.2	85.2		NED		
12093	68	IDC	2	0	0	NEG	TN	IIIA	pT1cN2	71	2.4	7.48	4.7	NED	ADJ	AC-TXT
12349										92	3.1	137.1	14.6			
12944										1160	38.7	287.7	10.3			
13130										2263	75.4	268.9	17.0	NED		
7097	58	IDC	3	80	90	NEG	LLB	IIB	pT2N1a	73	2.4	45.64		NED	ADJ	AC-TXT
7343										95	3.2	93.88	11.6			
8157										178	5.9	108.9	18.6			
8666										1199	40.0	127.8	28.8			
9078										2369	79.0	57.27	18.7	NED		

SERUM CODE	AGE	Histology	G	ER (%)	PR (%)	HER2 /NEU	Molecular Type	Stage	TNM	Follow-up Days	Follow-up months	BF-09 µgE/ml	CA15.3 U/ml	STATUS	Treatment type	Regimen
8958	46	IDC	1	90	90	NEG	LLA	IIA	pT1N1	95	3.2	132.4		NED	ADJ	FEC
9315										116	3.9	41.82	6.7			
9739										1137	37.9	112.5	9.5			
10244										2772	92.4	71.45	11.8	NED		
8024	61	arcinosarcon	3	5	0	NEG	LLB	IA	pT1bN0	87	2.9	84.72	13.4	NED	ADJ	FEC
9150										1143	38.1	68.66	9.9			
9582										2737	91.2	74.21	14.8	NED		
14542	50	ILC	2	80	90	NEG	LLA	IB	pT1cN0	0	0.0	117.2		NED	Surgery	
14717										21	0.7	147.4	6.0			
14895										42	1.4	105.9			ADJ	FEC 75
15113										1064	35.5	122.5	6.5			
15378										2091	69.7	158	7.3	NED		
7729	44	IDC	1	90	90	NEG	LLA	IIA	T2N0	0	0.0	130.4	22.3	NED	ADJ	FEC 75
7940										21	0.7	64.08	32.1			
8325										42	1.4	74.9	34.0			
8837										1112	37.1	293.4	27.2			
9746										2782	92.7	26.3	22.2	NED		
3132	39	IDC	2	80	5	NEG	LLB	IIIA	pT2N2	89	3.0	150.3		NED	ADJ	EC-TxT
3495										119	4.0	73.13				
4058										161	5.4	115.9	23.68			
5098										1309	43.6	89.62	22.64	NED		
7679	61	IDC	3	0	0	NEG	TN	IA	pT1N0	69	2.3	59.83	8.7	NED	ADJ	FEC 75
7891										91	3.0	0	6.2			
9099										153	5.1	30.69	7.4			
9511										2211	73.7	92.18	8.7	NED		
10533	50	IDC	1	95	95	NEG	LLA	IA	pT1N0	64	2.1	228.2	20.7	NED	ADJ	TAM + Enantone
10592										68	2.3	210.1	19.1			
10951										97	3.2	340	20.6			
11676										1934	64.5	60.95	21	NED		
6323	61	IDC	2	90	50	NEG	LLA	IIA	pT2N0	99	3.3	68.18	32.7	NED	ADJ	FEC
6593										121	4.0	142.4	27			
7145										191	6.4	135.6	34.7			CMF
7392										224	7.5	205.3	29			
7982										2304	76.8	129.8	32.9	NED		
24492	41	IDC	2	90	80	POS	LLB	IIIB	cT4N0	39	1.3	18.3	13.5	NED	ADJ	FEC
136805										629	21.0	39.58	12.8			
28223										901	30.0	18.300	11.5	NED		
16963	43	IDC	2	90	0	NEG	LLB	IA	pT1cpN0	59	2.0	148.4		NED	Hormonal	
18578										249	8.3	227.9	14.3			
93204										1675	55.8	219	16.1	NED		
9064	39	IDC	3	75	70	NEG	LLA	IIB	pT2pN1	96	3.2	29.55	37.4	NED	ADJ	EC-Taxotere
11727										286	9.5	60.81	23.0			
12040										317	10.6	48.83				
13448										463	15.4	99.02				
28342										2856	95.2	128.7	20.4	NED		
