# Supplementary material for: A Novel Protein Serum Biomarker Assay for Tracking (Neo)adjuvant and Metastatic Therapy Efficacy and Enabling the Timely Detection of Relapse in Breast Cancer
Source: Cancers (Basel). 2025 Dec 16;17(24):4004. doi: 10.3390/cancers17244004 (PMC12731775; doi:10.3390/cancers17244004)
Supplement: Supplementary file 1 [file cancers-17-04004-s001.zip › Supplementary Data S2.pdf]

Sample #	Disease	BF9 Conc.µgE/ml
1	normal	8.143
2	normal	33.05
3	normal	4.598
4	normal	13.4
5	normal	4.82
6	normal	2.585
7	normal	11.04
8	normal	10.25
9	normal	47.34
10	normal	8.478
11	normal	39.81
12	normal	17.74
13	normal	86.01
14	normal	25.98
15	normal	27.9
16	normal	4.313
17	normal	35.51
18	normal	4.18
19	normal	17.56
20	normal	6.326
21	normal	6.478
22	normal	8.596
23	normal	7.471
24	normal	8.032
25	normal	4.627
26	normal	4.34
27	normal	1.557
28	normal	11.7
29	normal	0.7079
30	normal	13.03
31	normal	21.87
32	normal	3.075
33	normal	106.1
34	normal	17.67
35	normal	7.556
36	breast-benign disease	2.516
37	breast-benign disease	0.798
38	normal	10.87
39	normal	4.051
40	breast-benign disease	11.11
41	breast-benign disease	62.76
42	normal	17.02
43	normal	84.05
44	breast-benign disease	31.89
45	normal	34.13
46	breast-benign disease	32.62
47	breast-benign disease	20.01
48	normal	3.075
49	normal	17.83
50	normal	7.228
51	breast-benign disease	102.2
52	breast-benign disease	4.283
53	breast-benign disease	434.6
54	breast-benign disease	23.38

90th percentile: 57 uge/ml
95th percentile: 86 uge/ml

55	normal	2.904
56	breast-benign disease	17.17
57	breast-benign disease	6.817
58	breast-benign disease	32.93
59	normal	27.09
60	normal	0.7984
61	normal	21.04
62	normal	6.27
63	normal	5.611
64	breast-benign disease	15.91
65	breast-benign disease	349.6
66	breast-benign disease	84.11
67	normal	19.8
68	normal	4.213
69	breast-benign disease	149
70	breast-benign disease	56.02
71	normal	7.199
72	normal	4.597
73	normal	19.99
74	breast-benign disease	10.53
75	normal	15.87
76	breast-benign disease	0.5294
77	breast-benign disease	4.975
78	normal	1.81
79	normal	6.606
80	normal	1.976
81	normal	1.456
82	normal	7.123
83	normal	0.707
84	breast-benign disease	3.52
85	breast-benign disease	68.93
86	breast-benign disease	39.3
87	normal	10.53
88	normal	0.707
89	breast-benign disease	0.798
90	normal	30.91
91	breast-benign disease	4.15
92	normal	11.71
93	breast-benign disease	23.28
94	normal	7.613
95	normal	6.703
96	breast-benign disease	0.798
97	breast-benign disease	1.181
98	normal	9.153
99	normal	2.033
100	normal	9.854
101	breast-benign disease	11.28
102	normal	16.19
103	normal	10.63
104	normal	10.3
105	normal	9.41
106	normal	55.91
107	breast-benign disease	2.64
108	normal	52.41
