# Supplementary material for: A Novel Protein Serum Biomarker Assay for Tracking (Neo)adjuvant and Metastatic Therapy Efficacy and Enabling the Timely Detection of Relapse in Breast Cancer
Source: Cancers (Basel). 2025 Dec 16;17(24):4004. doi: 10.3390/cancers17244004 (PMC12731775; doi:10.3390/cancers17244004)
Supplement: Supplementary file 1 [file cancers-17-04004-s001.zip › Supplementary Data S3.pdf]

PATIENT ID	Time from Sx/Tx start (months)	BF-09	Patient Status
**S01**	**0**	57.7	BSL before PD*
**S06**	**0**	109.1	BSL before PD*
**S08**	**3.3**	79	BSL before PD*
**S10**	**4.5**	57.1	BSL before PD*
**S12**	**3.5**	37.4	BSL before PD*
**S04**	**49.8****	90.5	BSL before PD*
**S43**	5.6	109.6	BSL before PD*
**S15**	**7**	100.2	BSL before PD*
**S05**	**3**	86.4	BSL before PD*
**S02**	**33.9****	18	BSL before PD*
**S03**	**41***	58.2	BSL before PD*
**S73**	**1.7**	44.3	BSL before PD*
**S07**	**4.2**	91.4	BSL before PD*
**S74**	**33.4****	49.3	BSL before PD*
**S11**	**2.2**	40.6	BSL before PD*
**S14**	**4.6**	96.2	BSL before PD*
**S32**	**89.6**	0	NED at last FU
**S22**	**92.7**	26.3	NED at last FU
**S31**	**64.9**	26.79	NED at last FU
**S18**	**70**	57.27	NED at last FU
**S25**	**64.5**	60.95	NED at last FU
**S19**	**92.4**	71.45	NED at last FU
**S33**	**57.5**	71.98	NED at last FU
**S20**	**91.2**	74.21	NED at last FU
**S16**	**92.2**	85.2	NED at last FU
**S24**	**73.7**	92.18	NED at last FU
**S29**	**95.2**	128.7	NED at last FU
**S26**	**76.8**	129.8	NED at last FU
**S35**	**107.5**	151.2	NED at last FU
**S21**	**69.7**	158	NED at last FU
**S39**	**109.8**	209.6	NED at last FU
**S28**	**55.8**	219	NED at last FU
**S17**	**75.4**	268.9	NED at last FU
**S27**	**30*****	18.3	NED at last FU
**S23**	**43.6*****	89.62	NED at last FU
**S38**	0	102.7	CR after NAT
**S45**	0	108.4	CR after NAT

153.9 90th percentile

*** BSL within 1 Yr from Sx/AT start**

**** 1st or close to 1st datapoint available**

***** last point available**
