# Supplementary material for: A Novel Protein Serum Biomarker Assay for Tracking (Neo)adjuvant and Metastatic Therapy Efficacy and Enabling the Timely Detection of Relapse in Breast Cancer
Source: Cancers (Basel). 2025 Dec 16;17(24):4004. doi: 10.3390/cancers17244004 (PMC12731775; doi:10.3390/cancers17244004)
Supplement: Supplementary file 1 [file cancers-17-04004-s001.zip › Supplementary Data S4.pdf]

Data used to calculate EDSA-BC assay %CV

BF-09 Level 1	Rep 1	Rep 2	Day Mean
Day 1	43.99	39.26	41.625
Day 2	51.45	44.69	48.07
Day 3	38.88	45.01	41.945
Day 4	42.04	38.27	40.155
Day 5	45.04	45.43	45.235
Day 6	36.51	44.06	40.285
Day 7	38.7	34.75	36.725
Day 8	46.43	39.63	43.03

BF-09 Level 2	Rep 1	Rep 2	Day Mean
Day 1	142.3	153.1	147.7
Day 2	157.9	146	151.95
Day 4	116.9	116.6	116.75
Day 5	114	138.2	126.1
Day 6	133.5	119.4	126.45
