# Supplementary material for: A Novel Protein Serum Biomarker Assay for Tracking (Neo)adjuvant and Metastatic Therapy Efficacy and Enabling the Timely Detection of Relapse in Breast Cancer
Source: Cancers (Basel). 2025 Dec 16;17(24):4004. doi: 10.3390/cancers17244004 (PMC12731775; doi:10.3390/cancers17244004)
Supplement: Supplementary file 1 [file cancers-17-04004-s001.zip › Supplementary Materials.pdf]

Supplementary Materials for

A novel protein serum biomarker assay for tracking (neo)adjuvant and metastatic therapy efficacy and enabling the timely monitoring of relapses in breast cancer

Jeffrey Dea [1,†], Christine Chavany [1,†], Rosaura P. C. Valle [1], Rafael Hernández González [2,‡] and Moncef Jendoubi [1,*]
Corresponding author: moncefj@milagen.com

Contents:

Supplementary Methods
Tables S1 to S10
Figure S1
Excluded patient analysis

Other Supplementary Materials submitted as separate file:

Data S1
Data S2
Data S3
Data S4

Supplementary Methods

Within laboratory Precision.

The study was performed by a single operator, at a single site. Two Breast cancer patient serum pools (Level 1 and Level 2) were run in two replicates per run, 1 run per day, over the course of up to 8 days. The data were analyzed for repeatability (within-run), between days, and within-laboratory precision.

Within Run SD (Sr) was calculated as, $Sr = \sqrt{\frac{\sum_{i=1}^{I}(xi1-xi2)^2}{2I}}$

I= total number of days; xi1= result for replicate 1 on day I; xi2= result for replicate 2 on day i

Between Run SD (B) was calculated as; $B = \sqrt{\frac{\sum_{i=1}^{I}(Xi-Xt)^2}{I-1}}$

I= number of days; Xi=average replicates on day I; Xt= average of all results over all days

Within laboratory SD (St) was calculated as; $St = \sqrt{\frac{\mathrm{N}-1}{\mathrm{N}} \times Sr^2 + B^2}$

N= number of replicates per run; B between SD; Sr within run SD

%CV was calculated as SD/Mean*100.

Total within laboratory %CV of the assay was calculated as the average of within laboratory %CV over the two levels concentration.

Results are summarized below in **Table S2**. Total within laboratory %CV for the assay (average of level 1 & Level 2) was calculated as 10.7%.

Identifying Significant Percent-Change Value for Assay Positivity/Negativity

ROC analysis of the total set (72 patients, n=99 events) was performed using percent change in BF-09 biomarker associated with progression/no progression events (Table 2). The following table summarizes various sensitivity values and the resulting specificities and cut-off values at a chosen sensitivity for the assay. Using a threshold of 30% change, the sensitivity of the BF-09 assay is 74.2% (CI 62-84.2%) at a specificity of 84.8% (CI 68.1-94.9%) (Table 2, S3). A 30% change value (defined as an increase or decrease in value that is at least 30% greater than the previous value of the test) is at least 2.5 times the total CV% of the assay (10.7%) tested at two concentrations (level 1 & 2) that span the range of detection. This was therefore chosen to ensure the assay change value would not be attributed to assay variation.

Table S1.

Characteristics		
**Median age at diagnosis, year (range)**	51	28-75
**Estrogen receptor status, N (%)**		
Positive	63	74.1
Negative	22	25.9
**Progesterone status, N (%)**		
Positive	60	70.6
Negative	25	29.4
**HER2 status, N (%)**		
Positive	14	16.5
Negative	71	83.5
**Molecular type, N (%)**		
Luminal-A	25	30.9
Luminal-B	41	48.2
Triple Negative	16	18.8
HER2-positive	3	3.5
**Histology at diagnosis, N (%)**		
Invasive Ductal Carcinoma	73	85.9
Invasive Lobular Carcinoma	9	10.6
Other	3	3.5
**Tumor stage, N (%)**		
I	8	9.4
II	21	24.7
III	28	32.9
IV	28	32.9
**Grade, N (%)**		
1	5	5.9
2	35	41.2
3	41	48.2
Unknown	4	4.7
**Treatment, N (%)**		
NAT	14	16.5
AT	16	18.8
MBCT	30	35.3
NAT+ AT	8	9.4
NAT± AT& MBCT	4	4.7
AT + MBCT	13	15.3

Table S1: Clinicopathological characteristics of patients at baseline. Full set of 85 patients. Abbreviations: Neoadjuvant therapy (NAT), Adjuvant therapy (AT), metastatic breast cancer therapy (MBCT), number of patients (N).

Table S2.

	Total Mean (µgE/ml)	Within Run SD	Within Run %CV	Daily Means SD	Daily Means % CV	Within laboratory SD	Within laboratory % CV
Level 1	42.1	2.7	6.5	3.4	8.1	3.9	9.3
Level 2	133.8	7.2	5.4	15.2	11.4	16.1	12.0

Table S2. Assay precision and variability. Mean concentration, standard deviation (SD) and percent coefficient variation (%CV) of assay variability are shown for two breast cancer patient serum pools (Level 1 and 2).

Table S3.

Cutoff BF-09	SE (%)	CI -SE (%)	SP (%)	CI-SP (%)
>13.7%	74.2	62.0-84.2	78.8	61.1-91.0
>30.0%	74.2	62.0-84.2	84.8	68.1-94.9
>38.1%	72.7	60.4-83	87.9	71.8-96.6
>43.9%	68.2	55.6-79.1	87.9	71.8-96.6
>51.4%	66.7	54.0-77.8	87.9	71.8-96.6
>62.9%	65.1	52.4-76.5	90.9	75.7-98.1

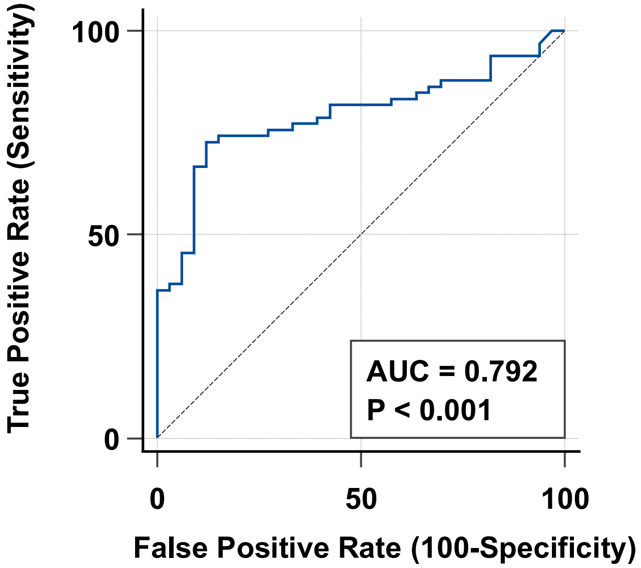

Table S3. Sensitivity and Specificity at different values of BF-09 percent change. ROC analysis was done to analyze percent changes in BF-09 in association with progression/non-progression events in order to find ideal percent change Values. Sensitivities for progression, specificities for non-progression and 95% CI are shown for different percentage changes.

Table S4.

Phase of treatment	Sensitivity (%)	Specificity (%)
Cutoff: 57 µgE/mL (90th percentile of non-cancer patients)		
**NAT**	80.0	14.3
**AT**	81.3	21.1
**MBCT**	63.3	-
Cutoff: 86 µgE/mL (95th percentile of non-cancer patients)		
**NAT**	40.0	21.4
**AT**	56.3	52.6
**MBCT**	50.0	-
Cutoff: 153.9 µgE/mL (90th percentile CR/NED)		
**NAT**	20.0	78.6
**AT**	37.5	78.9
**MBCT**	23.3	-

Table S4. Sensitivity and Specificity of EDSA-BC when using static cutoffs. Sensitivity for progressive disease or relapse and specificity for partial remission or NED given at neoadjuvant therapy (NAT), post-adjuvant surveillance (AT) and metastatic breast cancer therapy (MBCT) at three different cutoffs. Specificity for MBCT not calculated as only patients who had partial remission did so during neoadjuvant therapy so were counted as part of NAT. Points of clinical assessment of progressive disease/partial remission/NED/MET were used for analysis. Non-cancer patients were from a previous study and are shown in Supplementary Data S2.

Table S5.

						Regimen	
**Fig. 1**	**Pt #**	**Subtype**	**Histology**	**Grade**	**Stage**	**NAT**	**AT**
**C**	S50	LLB	IDC	2	IIIA	AI	
**D**	S44	LLB	IDC	3	IIIA	AC-TXT	
**E**	S39	LLA	IDC	2	IIA	FEC100	FEC100+ TXT
**F**	S31	HR+/ HER2+	IDC	1	IIIB	PTX-HERC	AI

Table S5. Summary of representative patient characteristics during NAT treatment follow-up. Summary of patient characteristics depicted in Figure 1 includes molecular subtype, histology, grade (G), BC stage, Neoadjuvant therapy (NAT) and Adjuvant therapy (AT) regimen, as applicable. **Abbreviations used**: invasive ductal (IDC), luminal-A (LLA), luminal-B (LLB), hormone receptor-positive/HER2-positive (HR+/HER2+) BC subtypes, aromatase inhibitor (AI), doxorubicin, cyclophosphamide (AC), Paclitaxel (PTX), fluorouracil, epirubicin, cyclophosphamide (FEC), taxotere (TXT), Paclitaxel (PTX), Herceptin (HERC), doxorubicin, cyclophosphamide followed by Taxotere (AC-TXT).

Table S6.

Sensitivity by Stage		
**Stage**	**Number EDSA-BC positive out of total**	**Sensitivity (%)**
I	3/3	100
II	2/3	66.7
III	7/10	70
**Sensitivity by Subtype**		
**Subtype**	**Number EDSA-BC positive out of total**	**Sensitivity (%)**
HR+/HER2-	5/5	100
TNBC	5/7	71.4
HR+/HER2+	1/3	33.3
HER2+	1/1	100
**Sensitivity of Secondary vs Distant Relapse**		
**Location**	**Number EDSA-BC positive out of total**	**Sensitivity (%)**
Secondary Cancer	2/2	100
Distant	10/14	71

Table S6. Sensitivity of AT surveillance by stage, subtype and relapse location. EDSA-BC sensitivity in detecting post-adjuvant recurrences in different categories. Hormone-receptor positive (HR+) includes estrogen receptor (ER) and/or progesterone receptor (PR) positive breast cancers. Triple negative breast cancer (TNBC).

Table S7.

						REGIMEN	
**Fig. 2**	**Pt #**	**Subtype**	**Histology**	**Grade**	**Stage**	**AT**	**(I-III)- line**
**C**	S12	LLB	IDC		IIIB	(1) FEC;<br>(2) Hormone/ Radiotherapy	
**D**	S02	TN	IDC	3	IIIC	TC	Capecitabine (I); TC (II)
**E**	S25	LLA	IDC	1	IA	FEC	
**F**	S20	LLB	CARS	3	IA	FEC	

Table S7. Summary of representative patient characteristics undergoing adjuvant therapy treatment follow up. Summary of patient characteristics depicted in Figure 2 includes molecular subtype, histology, grade (G), BC stage and adjuvant therapy (AT), and metastatic breast cancer therapy (I- III- line) regimen, as applicable. **Abbreviations used**: invasive ductal (IDC), carcinosarcoma (CARS), luminal A (LLA), luminal-B (LLB), triple negative (TN) BC subtypes, fluorouracil, epirubicin, cyclophosphamide (FEC), Taxotere, cyclophosphamide (TC).

Table S8.

Location of recurrence	N	Detected (ratio)	Detected (%)
Bone	11	10/11	90.9
Lung	7	5/7	71.4
Liver	15	11/15	73.3
Nodes	6	5/6	83.3
Local	4	4/4	100
Bone + othe sites (Lung/Nodes / Brain/Liver)	7	4/7	57.1
Lung + Liver or Nodes	5	3/5	60
Brain	1	0/1	0
**Total**	**56**	**42/56**	**75**

Table S8. Per sample sensitivity of AT and MBCT patients by relapse site. Counts of how many relapses by site observed in this cohort. Includes all metastatic time points from both AT and MBCT patients (n = 56 relapses from 39 patients). Also shown are ratio and percentages of how many recurrences detected by increases in BF-09 serum level over total number of relapses per site.

Table S9.

Fig. 4	Pt #	Subtype	Histology	Grade	Stage	Regimen	
						**I-line**	**II-line**
**C**	S64	LLB	IDC	2	IV	FEC+ Dexrazoxane	TXT-Capecitabine
**D**	S55	LLA	ILC	3	IV	PTX+BEV	VNR/ Capecitabine
**E**	S65	TN	IDC		IV	PTX+BEV	CP-PTX-BEV
**F**	S57	LLA	IDC	3	IV	PTX+BEV	Fulvestrant + Denosumab

Table S9. Summary of representative patient characteristics undergoing metastatic breast cancer treatment follow up. Summary of patient characteristics depicted in Figure 3 includes molecular subtype, histology, grade (G), BC stage and metastatic breast cancer therapy (I-II- line) regimen. **Abbreviations used**: invasive ductal (IDC), invasive lobular (ILC), luminal-A (LLA), luminal-B (LLB), triple negative (TN) BC subtypes, fluorouracil, epirubicin, cyclophosphamide (FEC), Paclitaxel (PTX), Bevacizumab (BEV), carboplatin (CP), taxotere (TXT), vinorelbine (VNR).

Table S10.

Therapy Type	Number EDSA-BC positive out of total	Sensitivity (%)	CI (%)
Taxane + other Chemotherapy	8/10	80	44.4-97.5
Anthracycline + other Chemotherapy (excluding taxanes)	8/9	88.9	51.8-99-7
Taxane + Targeted Therapy	17/19	89.5	66.9-98.7
Endocrine + Targeted therapy	4/6	66.7	22.3-95.7
Taxane only	1/6	16.7	0.4-64.1

Table S10. Sensitivity of EDSA-BC in AT & MBCT by therapy regimen type. Sensitivity for disease progression grouped by therapy regimen type.

Figure S1.

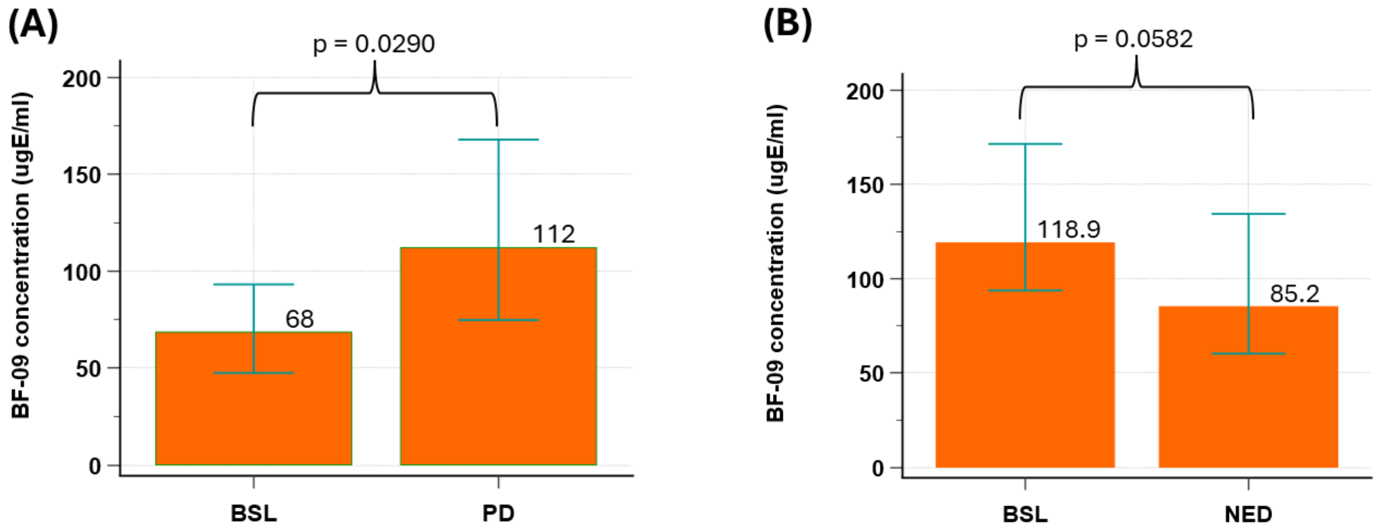

Figure S1: Comparison of median BF-09 serum levels after adjuvant treatment. Top of columns represent median serum levels of BF-09 between baseline (BSL) within 1 year of AT start (except 4 points) and **(A)** progressive disease (PD) and **(B)** non-evidence of disease (NED) at 6-year follow-up points (except for 2 patients who only had 3-year follow-ups). 95% confidence intervals for median represented by superimposed error bars. P-values calculated via Wilcoxon paired sample test.

Supplementary Text: Excluded patients' analysis

Four patients were excluded from the concordance analysis for lack of sufficient clinical information to interpret discrepancy between BF-09 levels and SOC disease status. Three are discussed below.

Patients **#S37** and **#S49** (both stage III patients, Supplementary Data S1) had levels of BF-09 that rose sharply at surgery while their clinical status was determined by histopathology as PR. CA15-3 levels stayed consistently at borderline level up to 1.6 months prior to surgery in patients **#S49**. In patient **#S37**, BF-09 level rose again (496%), 5.6 months after surgery and transition to adjuvant therapy (TC) with a concomitant rise of CA15-3 (31.9U/ml, 41%) contradicting a clinical assessment of NED. Patient **#S75** (stage II patient, Supplementary Data S1) is a similar case to patient **#S37**. While no other datapoints could confirm the progression of the disease, these data suggest persistence of residual disease in these patients.

Data S1. (separate file)

Patient Clinical information and EDSA-BC test results.

Data S2. (separate file)

BF-09 reference cutoff in non-cancer population based on 90th and 95th percentile.

Data S3. (separate file)

BF-09 reference cutoff in BC survivors' baselines based on 95th percentile.

Data S4. (separate file)

EDSA-BC assay %CV calculation dataset.
